# Are the Current Guidelines on Caffeine Use in Sport Optimal for Everyone? Inter-individual Variation in Caffeine Ergogenicity, and a Move Towards Personalised Sports Nutrition

**DOI:** 10.1007/s40279-017-0776-1

**Published:** 2017-08-29

**Authors:** Craig Pickering, John Kiely

**Affiliations:** 1 Institute of Coaching and Performance, School of Sport and Wellbeing, University of Central Lancashire, Preston, PR1 2HE UK; 2 Exercise and Nutritional Genomics Research Centre, DNAFit Ltd, London, UK

## Abstract

Caffeine use is widespread in sport, with a strong evidence base demonstrating its ergogenic effect. Based on existing research, current guidelines recommend ingestion of 3–9 mg/kg approximately 60 min prior to exercise. However, the magnitude of performance enhancement following caffeine ingestion differs substantially between individuals, with the spectrum of responses ranging between highly ergogenic to ergolytic. These extensive inter-individual response distinctions are mediated by variation in individual genotype, environmental factors, and the legacy of prior experiences partially mediated via epigenetic mechanisms. Here, we briefly review the drivers of this inter-individual variation in caffeine response, focusing on the impact of common polymorphisms within two genes, *CYP1A2* and *ADORA2A.* Contemporary evidence suggests current standardised guidelines are optimal for only a sub-set of the athlete population. Clearer understanding of the factors underpinning inter-individual variation potentially facilitates a more nuanced, and individually and context-specific customisation of caffeine ingestion guidelines, specific to an individual’s biology, history, and competitive situation. Finally, we identify current knowledge deficits in this area, along with future associated research questions.

## Key Points

**Table:** 

There is substantial variation between individuals when it comes to the performance improvement seen following caffeine ingestion in sport.
These differences are mediated, in part, by genetic variation between individuals.
Knowledge of this variation could lead to the development of improved caffeine usage guidelines for athletes.

## Introduction

1,3,7-Trimethylxanthine (caffeine) is one of the most widely used performance enhancing drugs. Between 1984 and 2004, caffeine was banned for in-competition use, although only at very high doses (12 μg mL^−1^). Nevertheless, this did not deter athletes, with research demonstrating that 74% of samples tested via the anti-doping process contained measurable levels of caffeine [1]. Since the removal of the ban, caffeine use has remained consistent, with measurable levels found in 74% of samples between 2004 and 2008 [2], illustrating that the use of caffeine is widespread in athletic populations.

The performance enhancing effects of caffeine have been known for over 100 years [3]. These effects are well replicated in both endurance-based activities [4] and repeated high-intensity efforts [5]. Similarly, caffeine appears to have a positive effect on muscular endurance [6–8], whereas its impact on maximum strength is less clear [9–11].

Caffeine exerts its ergogenic effect via several different proposed mechanisms. Within the central nervous system (CNS), caffeine acts as a competitive adenosine receptor antagonist [12], thereby reducing adenosine’s downregulation of arousal and nervous activity [13]. Additionally, the binding of caffeine to adenosine receptors increases neurotransmitter release and muscle firing rates [14]. Caffeine also stimulates adrenaline secretion [15], alters substrate utilisation [16], increases cellular ion release [17], and decreases pain perception [18, 19], all of which can improve exercise performance.

Elevated caffeine concentrations appear in the bloodstream as quickly as 15 min post-ingestion, peaking after about 60 min, with a 3- to 4-h half-life [15]. Caffeine is primarily metabolised in the liver, almost exclusively by cytochrome P450 enzymes, into paraxanthine, theophylline, and theobromine [20]; these in turn may mediate some of caffeine’s performance enhancing effects [15]. There remains the possibility that caffeine metabolism also occurs within the CNS, although this has been primarily studied in animal models [21]. There is also evidence of cytochrome P450 expression and activity within the CNS, raising the possibility that localised CNS caffeine metabolism is partially mediated by these enzymes [22]. However, overall, the pharmacokinetics of caffeine metabolism within the human CNS are poorly understood at present.

Typically, generalised guidelines recommend ingestion of 3–9 mg/kg of caffeine approximately 60 min prior to exercise, and suggest there are no additional benefits associated with higher doses [23–25]. However, recent research has illustrated that ergogenic effects of caffeine can occur with a wide variety of caffeine doses and timings. For example, a recent review [26] focused on the effects of low doses of caffeine (<3 mg/kg) on performance enhancement, finding that lower intakes of caffeine do tend to exert ergogenic effects. However, it is not clear whether these effects are equivalent to those seen with doses of 3 mg/kg or above. In relation to optimal timings of intake, Cox et al. [27] illustrated that 6 mg/kg of caffeine consumed 60 min prior to exercise was no more effective than six doses of 1 mg/kg of caffeine spread throughout the exercise bout. Accordingly, at least in some longer duration athletic events, caffeine ingestion during the event may be advisable. The prevalent use of caffeine within sport, and the assumed universal applicability of these generalised caffeine guidelines, seem to suggest there is a standard, predictable response to caffeine across individuals. Within this article, we discuss why this is not the case, and illustrate that, in fact, there is considerable inter-individual variation in the ergogenic effects of caffeine ingestion. We also identify the various interacting causes underpinning this diversity in inter-individual response. Finally, we propose potential research questions that, if answered, will facilitate the evolution of more personalised guidelines for caffeine use within sporting contexts.

## Inter-subject Variation in the Response to Caffeine

Whilst caffeine’s ergogenic effects are clear, the research findings demonstrating these benefits are conventionally calculated using the mean cohort responses. Crucially, these mean responses are considered an accurate estimation of the likely responses of each individual within the group. Yet numerous studies over the course of the past 2 decades illustrate the extent of individual variation commonly occurring subsequent to introduced interventions. The magnitude of this inter-individual response is well demonstrated in studies investigating individual fitness adaptation response to carefully controlled exercise interventions [28–30]. Is this also the case when it comes to the ergogenic effects of caffeine ingestion?

A small number of papers give us some insight into this question, either by directly studying the inter-subject variability in response to caffeine, or by publishing individual subject data. Jenkins et al. [31] compared the effects of low caffeine doses (1, 2, and 3 mg/kg) against placebo on a 15-min maximum cycle in 13 cyclists. The main finding was that caffeine improved mean performance by 3.9% (2 mg/kg) and 2.9% (3 mg/kg), respectively, versus placebo, with no improvements in the 1 mg/kg trial. This suggests that doses of 2 and 3 mg/kg are ergogenic for endurance performance. However, inspection of the individual data demonstrates large inter-individual variation in these effects. Most subjects exhibited large variations, with a performance decrement at some doses of caffeine, and performance enhancement at others. One subject, for example, did not demonstrate an ergogenic effect at any dose, whereas four subjects found caffeine ergogenic at all doses. Similarly, in a randomised, cross-over trial design, Graham and Spriet [32] put seven runners through treadmill and cycle ergometer exercise trials to exhaustion with either placebo or 9 mg/kg of caffeine. The caffeine dose significantly improved time to exhaustion for all subjects, but there was a large variation in the magnitude of this effect, with the caffeine trial lasting between 105 and 250% of the placebo trial. Other studies support this variation in ergogenic response to caffeine supplementation in individuals, with some individuals showing large improvements, and others no, or even negative, effects of caffeine supplementation [33, 34].

## Why Does this Individual Response Exist?

### The Genetics of Individual Variation in Caffeine Response

As with other complex phenotypes, individual responses following caffeine ingestion are polygenic phenomena, mediated by multiple interacting genes [35, 36]. This does not mean that it is impossible to determine the genetic drivers of individual differences, however. For example, habitual caffeine use is a highly complex trait, but genome-wide association studies have found single nucleotide polymorphisms (SNPs) associated with this behaviour [37]. Such findings indicate that, whilst genetic differences cannot explain all the variation, they can at least explain some. Below, we will examine variation within two genes that may impact caffeine ergogenicity, including a discussion regarding the mechanisms underlying this variation.

#### CYP1A2

The gene *CYP1A2* encodes cytochrome P450 1A2, an enzyme responsible for up to 95% of all caffeine metabolism [38]. A SNP within this gene, rs762551, affects the speed of caffeine metabolisation. Individuals with AA homozygotes (“fast metabolisers”) tend to produce more of this enzyme, and therefore metabolise caffeine more quickly. Conversely, C allele carriers (“slow metabolisers”) tend to have slower caffeine clearance [39]. The variable effects of this SNP are most well-established in regard to health, with myocardial infarction and hypertension risk increased in slow metabolisers consuming moderate (3–4 cups) amounts of coffee, whilst fast metabolisers exhibit a protective effect of moderate coffee consumption [40, 41].

These earlier medical studies prompted research into how the *CYP1A2* polymorphism might modify the ergogenic effects of caffeine. Womack et al. [42] put 35 trained male cyclists through two 40-km cycle time trials, following consumption of either 6 mg/kg of caffeine or placebo 60 min beforehand (Table 1). There was a significant effect of *CYP1A2* genotype on the ergogenic effects of caffeine, with AA genotypes (fast metabolisers) (4.9% improvement) seeing a significantly greater performance improvement than C allele carriers (slow metabolisers) (1.8% improvement). Within AA genotypes, caffeine improved performance by at least 1 min for 15 out of 16 subjects, whilst in C allele carriers only ten of 19 subjects saw an improvement greater than 1 min. These findings allowed the authors to conclude that caffeine has a greater ergogenic effect for *CYP1A2* AA genotypes than C allele carriers.

**Table 1:** Summary of published studies examining *CYP1A2* and *ADORA2A* polymorphisms and the ergogenic effect of caffeine on performance

Single nucleotide polymorphism	Study	Design	Sample characteristics	Caffeine dose	Measurement	Primary outcome
*CYP1A2* (rs762551)	Womack et al. [42]	Caffeine vs placebo	35 male recreationally competitive cyclists	6 mg/kg, 60 min prior	40-km cycle time trial	Caffeine reduced 40-km time trial time vs placebo by a greater (*p* < 0.05) magnitude in AA vs C allele carriers
	Klein et al. [47]	Caffeine vs placebo	16 collegiate male (*n* = 8) and female (*n* = 8) tennis players	6 mg/kg, 60 min prior	Maximal treadmill exercise test, tennis skills test	No significant impact of polymorphism on caffeine ergogenicity
	Pataky et al. [43]	Caffeine ingestion, placebo ingestion, caffeine mouth rinse, placebo mouth rinse	38 male (*n* = 25) and female (*n* = 13) recreational cyclists	6 mg/kg, 60 min prior, along with 25 mL of 1.14% caffeine mouth rinse	3-km cycle time trial	Greater performance enhancement in AC vs AA in both caffeine ingestion and caffeine rinse trials (no CC genotypes present)
	Algrain et al. [46]	Caffeine gum vs placebo	20 recreationally active males (*n* = 13) and females (*n* = 7)	300 mg caffeine gum, 10 min prior	15-min steady-state cycle, 10 min recovery, 15-min performance ride at 75% *V*O_2max_	No significant impact of polymorphism on caffeine ergogenicity
	Salinero et al. [48]	Caffeine vs placebo	21 recreationally active males (*n* = 14) and females (*n* = 7)	3 mg/kg	30-s Wingate test	No significant impact of polymorphism on caffeine ergogenicity
*ADORA2A* (rs5751876)	Loy et al. [54]	Caffeine vs placebo	12 females	5 mg/kg	20-min cycle at 60% *V*O_2max_, followed by 10-min maximum cycle	Total work increased for time trial genotypes following caffeine ingestion vs placebo. There were no improvements in the caffeine vs placebo trial for C allele carriers

*VO*_*2max*_ - maximal oxygen consumption

Since this initial paper, a small number of subsequent studies have been published. The same group published a paper hampered by a lack of CC genotypes, putting 38 recreational cyclists through four 3-km time trials under different experimental conditions: placebo mouth rinse + placebo ingestion, placebo mouth rinse + caffeine ingestion, caffeine mouth rinse + placebo ingestion, and caffeine mouth rinse + caffeine ingestion [43]. Both AC (4.1%) and AA (3.4%) genotypes saw performance improvements in the combined caffeine mouth rinse and ingestion trial, but only AC (6%) genotypes saw a performance improvement in the caffeine ingestion trial. The conclusion was that AC genotypes saw greater performance enhancement with caffeine ingestion, in contrast to Womack et al. [42]. One potential confounder identified by the authors was the shorter exercise trial duration (c.5 min) when compared to Womack et al. [42]. A second potential confounder is that Womack et al. [42] utilised trained subjects, whilst Pataky et al. [43] did not. Exercise appears to increase *CYP1A2* expression [44, 45], such that trained and untrained subjects may metabolise caffeine differently. Algrain et al. [46] reported no modifying effect of the *CYP1A2* polymorphism on the ergogenic effects of caffeine; however, they noted the small subject number (*n* = 20), the untrained status of these subjects, and the lower caffeine dose (approximately 255 mg). Klein et al. [47] and Salinero et al. [48] found no effect of the *CYP1A2* polymorphism on the effects of caffeine on tennis and Wingate test performance, respectively, although with modest sample sizes (*n* = 16 and 21).

Unpublished conference data presented by Guest and reported by Hutchinson [49] demonstrated that caffeine ingestion (4 mg/kg) improved 10-km cycle time trial performance by 1.2 min versus placebo in AA homozygotes; AC heterozygotes saw a 30-s improvement, whilst CC homozygotes saw a performance decrement of 2.5 min. Finally, Kingsley et al. [50] examined the interaction of caffeine (3 mg/kg) and *CYP1A2* genotype on a simulated soccer game. Whilst individual differences in caffeine response were evident, *CYP1A2* genotype did not explain this variation, potentially due to a lack of statistical power down to the low subject numbers (*n* = 10).

At present, the initial Womack et al. [42] paper has not yet been satisfactorily replicated, with some subsequent published research finding no impact of the *CYP1A2* polymorphism [46], or the opposite effect [43]. These subsequent papers have, however, tended to involve small sample sizes, be in untrained subjects, or be void of CC genotypes, present in approximately 10% of the population [39]. Further work is required to determine the full effect of this polymorphism on the ergogenic effects of caffeine on exercise.

#### ADORA2A

A SNP in the adenosine receptor gene *ADORA2A*, rs5751876, affects both habitual caffeine use [51] and sleep disturbances following caffeine use [52, 53]. Currently, only one pilot study has examined the effect of this SNP on the ergogenic effects of caffeine [54]. Twelve female subjects underwent a randomised, double-blinded, cross-over trial comprising two 10-min time trials following caffeine ingestion (5 mg/kg) or placebo. The TT homozygotes found caffeine ergogenic; the C allele carriers tended not to, with only one out of the six C allele carriers exhibiting an ergogenic effect. These subjects habitually consumed no caffeine or only low doses of caffeine (<250 mg/day), so it is not apparent how this might affect users habituated to higher doses. Subsequent research is required to replicate these findings, including within habitual caffeine users.

#### Potential Mechanisms: A Role for Caffeine Timing?

It is clear that genetic factors exert a large influence on individual responses to caffeine ingestion, even if these genetic factors have not yet been well elucidated. The mechanisms through which this genetic variation modifies caffeine ergogenicity are also unclear; regarding *CYP1A2,* it is speculated it could be due to a more rapid accumulation of caffeine metabolites in AA genotypes, which are hypothesised to potentially have a greater ergogenic effect than caffeine itself [42]. If correct, then caffeine timing becomes important; it might not be that C allele carriers find caffeine less ergogenic, just that it requires longer for caffeine to be metabolised to its ergogenic metabolites. Given caffeine’s many different mechanisms of action, it is likely each mechanism has polymorphisms that modify the ergogenic effect. For example, as caffeine reduces exercise-induced pain [18], SNPs related to pain tolerance could impact this effect. Similarly, genetic variation in adenosine receptors (such as polymorphisms within *ADORA2A*) is similarly promising. In the pilot study carried out by Loy et al. [54], there were a number of mechanisms proposed by the authors through which *ADORA2A* variation might affect caffeine ergogenicity, including enhanced motivation and motor unit recruitment in TT homozygotes.

#### Indirect Impact of Genetic Variation on Exercise Performance

Genetic variation also likely impacts exercise performance indirectly. Thomas et al. [55] examined the modifying effects of the *CYP1A2* polymorphism on recovery from exercise. Whilst overall there was no effect of the polymorphism on cardiac markers of recovery, there were significant differences in the square root of the mean of squared differences between successive R intervals (RMSSD) in heart rate variability monitoring. Similarly, polymorphisms within *ADORA2A* can predispose individuals to increased anxiety following caffeine ingestion [56, 57]. This is potentially of interest in individuals who suffer from pre- and within-competition anxiety, but also to individuals who may benefit from elevated levels of pre-competition arousal. *ADORA2A* polymorphisms are also associated with increased sleep disturbances following caffeine ingestion [53], which could impact individuals involved in evening competitions, or those involved in tightly spaced consecutive day competitions; here, sleep disturbances could significantly negatively impact exercise recovery.

### Environmental Factors Affecting Caffeine Response

There are also a variety of different non-genetic factors that can impact caffeine ergogenicity, many of which are controlled for in research. These include habitual use of caffeine, with habitual use assumed to potentially reduce the ergogenic effect of caffeine [58–60], although this finding is equivocal [61, 62]; perhaps habitual users simply require higher doses of caffeine to maintain the ergogenic effect. Other non-genetic factors affect caffeine metabolisation speed, often by increasing cytochrome P450 activity. These include smoking [63, 64], dietary vegetable intake [65], oral contraceptive use [66, 67], pregnancy [68], menstrual cycle stage [69], training status [44, 45], and hormone replacement therapy [70]. Other non-genetic, but controllable, factors affecting caffeine ergogenicity are related to the nature of caffeine ingestion, including caffeine dose [71], source [72–74], age [75], timing [76], time of day [76, 77], and training status [78, 79].

Finally, expectancy effects influence caffeine response. Saunders et al. [80] put subjects through time trials with either 6 mg/kg of caffeine, placebo, or control (neither caffeine nor placebo). Correct identification of caffeine ingestion gave a greater relative performance enhancement than the overall caffeine trial. Similarly, the belief that caffeine had been ingested in the placebo trial led to a likely beneficial effect. Correct identification of placebo led to possibly harmful effects, with some subjects showing a performance decrement compared to the control trial. This mirrors results of earlier research on the expectancy effect of caffeine. For example, Beedie et al. [81] showed that placebo caffeine ingestion improved endurance cycle performance in a dose-response manner, with higher placebo doses leading to greater performance improvements. Similarly, Pollo et al. [82] demonstrated that belief of caffeine ingestion improved time to fatigue in a maximal quadriceps extension task. When subjects are informed they have ingested caffeine, it appears to improve performance, even if they have been deceptively administered a placebo [80, 83].

It is important to consider that genetics also modify these environmental factors. For example, habitual caffeine use itself has a genetic underpinning [84], and certain genotypes appear to be more sensitive to the effects of placebo [85].

### Epigenetic Modifiers of Caffeine Response

Epigenetics refers to changes in gene function that occur without a change in nucleotide sequence [86]. Such changes can be heritable, but also modifiable over time within an individual [87]. Caffeine use undoubtedly induces epigenetic modifications [88–90], and these epigenetic modifications can impact caffeine clearance by altering CYP1A2 activity [91, 92]. However, it is not entirely clear how this might impact caffeine’s ergogenic effects. Long-term caffeine use potentially leads to habituation through both increased caffeine clearance (mediated by epigenetic modifications on cytochrome P450 genes [91]) and a decrease of excitability caused by caffeine—possibly via inhibition of genes affecting the dopaminergic and adenosine pathways [93]. Further research is required to establish the effects of epigenetics on the ergogenic effects of caffeine.

### “Non-responder” Versus “Did Not Respond”

Clearly, the individual response to caffeine is complex and subject to genetic, non-genetic (i.e. environmental), and epigenetic influences. Given that both environmental and epigenetic influences are not stable across time, an individual’s response to caffeine will vary. A clear example of this is that of habituation, briefly discussed in Sect. 3.2. In this context, regular use of caffeine may modify the ergogenic effects of caffeine at a particular dose. Beaumont et al. [59] illustrated that regular intakes of 3 mg/kg of caffeine daily attenuated the ergogenic effects of a pre-exercise dose of 3 mg/kg. Conversely, de Souza Gonçalves et al. [61] showed that habitual daily caffeine intakes of 350 mg/day were insufficient to reduce the ergogenic effects of 6 mg/kg of caffeine. This indicates that it is perhaps important that the pre-exercise caffeine dose exceeds the level of habitual intakes. So, whilst an individual might initially find a caffeine dose of 3 mg/kg ergogenic, if they then habitually consume 3 mg/kg of caffeine per day, this ergogenesis may be attenuated. As such, in an initial trial, the subject would be labelled as a caffeine “responder”, whilst in the subsequent trial, they would be labelled a “non-responder”. Such labels are becoming common place when reporting on inter-individual response to a stimulus. However, recent work [94] indicates that non-response to exercise can be reduced by changing training variables. We suggest the same is likely true for caffeine. As such, perhaps a more reflective characterisation would be to state that a subject “did not respond” to a particular intervention, as opposed to labelling them a “non-responder” [95], as this non-response may not occur were the intervention to be repeated and/or modified.

## Conclusions: What Next?

Academic studies have repeatedly demonstrated a performance enhancing effect of caffeine ingestion [4–6, 15]. Yet, simultaneously, this ergogenic response shows considerable inter-individual variation [31, 32]. This variation occurs via numerous factors, many of which are influenced by genetic predispositions [42, 54]. Although these individual responses are undoubtedly complex and subject to various modifying factors, the possibility remains that practitioners can glean sufficient partial insights to personalise caffeine intake. Polymorphisms in genes affecting caffeine metabolisation speed (*CYP1A2*) [42] and nervous system excitability (*ADORA2A*) [54] appear to have a directly modifying impact on the ergogenic effects of caffeine. Given the number of mechanisms through which caffeine appears to exert its action, it could be speculated that a variety of other polymorphisms will also have a contributing role. Recent developments in genetic profiling technology and more widespread access to, and affordability of, such technology raises the possibility that such insights may soon be readily available to sporting populations. This information could potentially be paired with knowledge of individual variation in other factors, such as circadian rhythm [76, 77], habitual caffeine use [58–60], medication intake [66, 67], and expectancy [80, 81, 83], all of which also impact the magnitude of performance enhancement seen after caffeine ingestion.

**Fig. 1:**
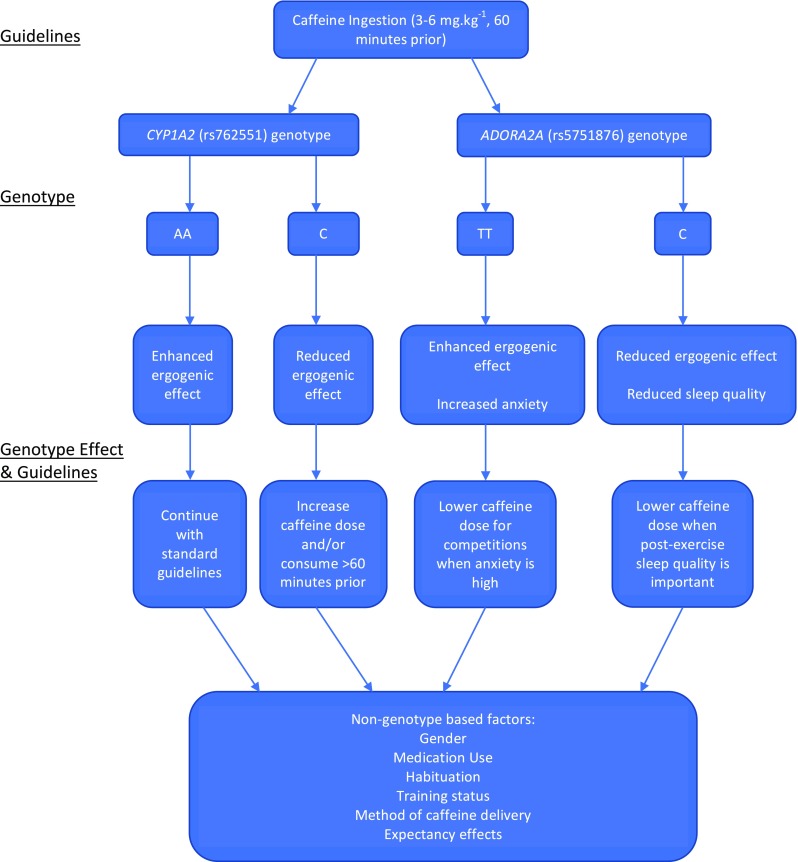
Genetic and non-genetic factors influencing caffeine ingestion decisions

Figure 1 summarises the genetic and non-genetic factors influencing caffeine ingestion decisions. Working from the top, the current best-practice guidelines are applied to different genotypes of genes identified to impact caffeine response. Based on the current evidence, genotype-based guidelines are then produced. Finally, these genotype guidelines must then be interpreted in the context of non-genetic factors, such as habitual use, to create individualised caffeine guidelines. As *CYP1A2* and *ADORA2A* polymorphisms have not yet been studied together, the potential interacting effects of these polymorphisms are currently unknown. Finally, the recommendations themselves are somewhat speculative, and further research is required to elucidate best practice in this area.

These individualised caffeine guidelines could also vary depending on the timing and importance of the competition. Given that genetic variation can modify sleep disturbances after caffeine ingestion [53], individuals more likely to suffer from these disturbances might consume less caffeine for an evening competition than a morning competition. This would be especially important if there were a number of competitions in close proximity, whereby reduced recovery following initial caffeine dose may impact subsequent exercise performance. Genetic variation can also impact feelings of anxiety following caffeine ingestion [56, 57]. This creates the possibility that certain genotypes should consume less caffeine for competitions where anxiety is likely to be higher, such as the Olympic Games or World Cup final, and more for competitions where anxiety will be lower, such as a league match.

This spawns an interesting situation; whilst caffeine is ergogenic, the current generalised guidelines of 3–9 mg/kg, 60 min prior [23–25] are clearly not optimal for everyone. What is not clear, however, is what these guidelines should be. Being able to develop more precise, individualised guidelines would be beneficial, especially given the prevalent caffeine use in elite sports. To enhance the advice given to athletes regarding caffeine use, a number of different questions will need to be answered:

1. Can the existing research on *CYP1A2* and *ADORA2A* be replicated, and can other genes that modify caffeine ergogenicity be identified?
2. Are there different optimal dosages and timing strategies for different genotypes?
3. Does caffeine habituation occur differently across genotypes?
4. Does the individual’s sex further alter the modifying aspect of genotype on caffeine ergogenicity?

By answering these questions and creating personalised caffeine guidelines, athletes will be able to fully maximise the performance enhancing effects of caffeine in a way that is matched to their unique biology. In addition, the awareness from coaches and athletes that sizeable variation exists in the response to caffeine ingestion may encourage them to be more experimental and flexible in the evolution of their caffeine strategies.
